# Supplementary material for: Agl24 is an ancient archaeal homolog of the eukaryotic N-glycan chitobiose synthesis enzymes
Source: eLife. 2022 Apr 8;11:e67448. doi: 10.7554/eLife.67448 (PMC8993221; doi:10.7554/eLife.67448)
Supplement: Supplementary file 1. — DELTA BLAST was performed to identify homologs of MurG (WP_063074721.1), Alg13 (NP_011468.1), Alg14(NP_009626.1), and Alg14-13 fusion in Sulfolobus acidocaldarius. [file elife-67448-supp1.pdf]

Description	Max score	Total score	Query cover	E value	Per. Ident	Accession	Saci gene	KO	Annotation
**MurG** WP_063074721.1 undecaprenyldiphospho-muramoylpentapeptide beta-N-acetylglucosaminyltransferase [*Escherichia coli*]									
conserved Archaeal protein [Sulfolobus acidocaldarius DSM 639]	77.0	77.0	76%	6e-17	14.52%	AAY81210.1	*saci_1907*	MW061 BM-A045-B027	RFaB GT1_YqgM_like
conserved protein [Sulfolobus acidocaldarius DSM 639]	71.2	71.2	98%	5e-15	16.76%	AAY80607.1	*saci_1262*	*essential*	MurG
hypothetical protein Saci_1921 [Sulfolobus acidocaldarius DSM 639]	71.2	71.2	72%	6e-15	13.33%	AAY81224.1	*saci_1921*	MW069 BM-A055-B003	RfaB
RecName: Full=Archaeal glycosylation protein 16 [Sulfolobus acidocaldarius DSM 639]	65.9	65.9	75%	3e-13	9.90%	Q4JAK2.1	*saci_0807*	MW043 BM-A110-B008	Archaeal glycosylation protein 16
partially conserved Archaeal protein [Sulfolobus acidocaldarius DSM 639]	61.2	61.2	96%	1e-11	12.76%	AAY81207.1	*saci_1904*	MW077 BM-A050-B002	Cell wall/membrane/envelope biogenesis
glycosyl transferase [Sulfolobus acidocaldarius DSM 639]	59.7	98.2	74%	5e-11	14.02%	AAY80548.1	*saci_1201*	MW053 BM-A150-B1	Glycogen synthase
glycosyl transferase group 1 [Sulfolobus acidocaldarius DSM 639]	47.8	47.8	77%	3e-07	12.38%	AAY81133.1	*saci_1827*	MW042 BM-A280-B001	GT1_Trehalose_phosphorylase
gylcosyl transferase group 1 protein [Sulfolobus acidocaldarius DSM 639]	45.8	45.8	49%	1e-06	16.59%	AAY80595.1	*saci_1249*	MW054 BM-A155-B001	GT1_YqgM_like
conserved protein [Sulfolobus acidocaldarius DSM 639]	42.4	42.4	34%	1e-05	18.11%	AAY80235.1	*saci_0869*	*essential*	Glycosyltransferase involved in cell wall biosynthesis
conserved protein [Sulfolobus acidocaldarius DSM 639]	42.4	42.4	58%	2e-05	9.91%	AAY81219.1	*saci_1916*	MW066 BM-A024-B001	
RecName: Full=UDP-sulfoquinovose synthase [Sulfolobus acidocaldarius DSM 639]	40.4	40.4	26%	7e-05	19.39%	Q4JBJ3.1	*saci_0423*	MW039 BM-A083-B008	Agl3 UDP-sulfoquinovose synthase
hypothetical protein Saci_1923 [Sulfolobus acidocaldarius DSM 639]	39.3	39.3	34%	1e-04	11.72%	AAY81226.1	*saci_1923*	n.d.	
conserved protein [Sulfolobus acidocaldarius DSM 639]	38.5	38.5	78%	3e-04	11.85%	AAY81225.1	*saci_1922*	MW070 BM-A060-B002	
**ALG13** NP_011468.1 N-acetylglucosaminyldiphosphodolichol N-acetylglucosaminyltransferase [*Saccharomyces cerevisiae* S288C]									
conserved protein [Sulfolobus acidocaldarius DSM 639]	43.9	43.9	58%	1e-06	17.36%	AAY80607.1	*saci_1262*		MurG
**ALG14** NP_009626.1 N-acetylglucosaminyldiphosphodolichol N-acetylglucosaminyltransferase [*Saccharomyces cerevisiae* S288C]									
	No homolog above the standard Delta-Blast e-value threshold of 0.05								
**Alg14-13 fusion** (NP_009626.1+ NP_011468.1)									
conserved protein [Sulfolobus acidocaldarius DSM 639]	59.7	59.7	73%	4e-11	16.92%	AAY80607.1	*saci_1262*		
