# Supplementary material for: Agl24 is an ancient archaeal homolog of the eukaryotic N-glycan chitobiose synthesis enzymes
Source: eLife. 2022 Apr 8;11:e67448. doi: 10.7554/eLife.67448 (PMC8993221; doi:10.7554/eLife.67448)
Supplement: Supplementary file 2. — Structural modelling was performed by SWISS-MODEL (Waterhouse et al., 2018), using either the full length Agl24 sequence or only the c-terminal Alg13-like part. Identified PDB numbers as well as the SWISS MODLE results, e.g.Global Model Quality Estimation (GMQE), quaternary structure quality estimate (QSQE), and sequence identity are shown. [file elife-67448-supp2.pdf]

PDB	RMSD	GMQE	QSQE	Identity	X-ray	Reference
Full Agl24 sequence						
3s2U MurG	13.82	0.51	-	17.2	2.2Å	(Brown et al., 2013)
1f0k MurG	15.94	0.55	0,31	17.36	1.9Å	(Ha et al., 2000)
1nlm MurG	16.22	0.55	0,20	17.36	2.5Å	(Hu et al., 2003)
Alphafold model	3.80	-	-	-	-	Supplement Data
c-terminal Agl13-like part of Agl24						
2ks6 Alg13	-	0.56	-	20.53	NMR	(Raman et al., 2010)
2jzc Alg13	-	0.56	-	19.87	NMR	(Wang et al., 2008)
1f0k MurG	-	0.46	0.17	19.70	1.9Å	(Ha et al., 2000)
1nlm MurG	-	0.46	-	19.70	2.5Å	(Hu et al., 2003)
