# Supplementary material for: Agl24 is an ancient archaeal homolog of the eukaryotic N-glycan chitobiose synthesis enzymes
Source: eLife. 2022 Apr 8;11:e67448. doi: 10.7554/eLife.67448 (PMC8993221; doi:10.7554/eLife.67448)
Supplement: Supplementary file 3. [file elife-67448-supp3.pdf]

Strains	Genotype	Reference
E. coli		
DH5α	F– ϕ80lacZΔM15 Δ(lacZYA-argF)U169 recA1 endA1 hsdR17(rK–, mK+) phoA supE44 λ– thi-1 gyrA96 relA1	(Taylor et al., 1993)
BL21 (DE3)	F^{-} *ompT hsdS*$_{B}$ (r$_{B}^{-}$, m$_{B}^{-}$) *gal dcm* (DE3)	(Studier and Moffatt, 1986)
ER1821	F- glnV44 e14-(McrA-) rfbD1? relA1? endA1 spoT1? thi-1 Δ(mcrC-mrr)114::IS10, MM294 background	(Meselson and Yuan, 1968)
Sulfolobus acidocaldarius		
MW001	*S. acidocaldarius* DSM 639, Δ*pyr*E	(Wagner et al., 2012)
BM-A305	*S. acidocaldarius MW001* with integrated deletion plasmid pSVA1312 incl. *pyr*EF	*This study*
BM-A306	*S. acidocaldarius MW001* with integrated deletion plasmid pSVA1312 incl. *pyr*EF	*This study*
BM-A719	*S. acidocaldarius MW001* with pSVA1337 (MAL$_{promotor}$ *saci1262,* C-terminal Strep- and His-Tag)	*This study*
BM-A720	*S. acidocaldarius MW001* with pSVA1339 (MAL$_{promotor}$ *saci1262*, N-terminal Strep- and His-Tag)	*This study*
BM-A724	*S. acidocaldarius MW001* with pBM-0078 (ARA$_{promotor}$ *saci1262* C-terminal Strep- and His-Tag)	*This study*
BM-A796	*S. acidocaldarius MW001* with pSVA1266 (Mal$_{promotor}$ *saci0423-agl3* C-terminal Strep- and His-Tag)	*This study*
**Plasmids for generating deletion mutants**		
pSAV0407	Gene targeting plasmid, pGEM-T Easy backbone, *pyrEF* cassette of *S. solfataricus*	(Wagner et al., 2012)
pSVA1266	Expression vector of *agl3* (*saci0423*) cloned into pSVA1450 with *Nco*I and *Eag*I	(Meyer et al., 2011)
pSVA1312	In-frame deletion of *agl24* (*saci1262*) cloned into pSVA407 with *Apa*I and *Bam*HI	*This study*
pSVA3338	Linear *saci1262*($_{short}$)-*pyrEF-saci1262* cut with *BamH*I and *Apa*I into pSVA407	*This study*
**Expression plasmids for *S. acidocaldarius***		
pSVA1481	*E. coli* vector with Ara-promoter and C-terminal Strep- and His-tag based on pGEM-T Easy backbone and pMZ1 cassette, pMZ: Amp^{r}, cloning vector containing replicon ColE1 (pBR322) and a 10X His-Strep tag sequence at the 3' end of the MC	Wagner & Albers
pSVA1518	Expression vector with ARA$_{promotor}$ and c-terminal Strep-His$_{10}$ tag	Wagner & Albers
pSVA1431	Expression vector with MAL$_{promotor}$ and c-terminal Strep-His$_{10}$ tag	Wagner & Albers
pSVA2301	Expression vector with MAL$_{promotor}$ and N-terminal His$_{10}$- Strep- tag	Wagner & Albers
pSVA1336	*agl24* cloned into pSVA1481 (ARA$_{promotor}$)with a C-terminal Strep- and His-tag with *Nco*I and *Pst*I	*This study*
pSVA1337	*agl24* with c-terminal Strep- and His-Tag derived from pSAV1336 subcloned into pSVA1431 (MAL$_{promotor}$) with *NcoI* and *EagI*	*This study*
pSVA1339	agl24 cloned into pSVA2301 with N-terminal His$_{10}$ Strep - TEV into pSVA2301 (MAL$_{promotor}$) with *NcoI* and *NotI* (Primer 4180 and 4181*)*	*This study*
pBM-0078	Ara promotor saci1262 with C-terminal Strep- and His-tag, cloned with *NcoI ApaI*	*This study*
**Expression plasmids for *E. coli***		
*p*Waldo	T7 expression vector containing TEV site-GFP-His$_{8}$	*(Waldo et al., 1999)*
pHD0499	pWaldo containg *Agl24*-GFP-His$_{8}$ cloned with *Xho*I and *Kpn*I	*This study*
pHD0554	pWaldo containg *Agl24*-H$_{14}$A-GFP-His$_{8}$ cloned with *Xho*I and *Kpn*I	*This study*
pHD0586	pWaldo containg *Agl24*-E$_{114}$A-GFP-His$_{8}$ cloned with *Xho*I and *Kpn*I	*This study*

Primer	Sequence (5´-3´)	Restriction Site
ΔAgl24		
4168	ACTA GGGCCC GGTCTTGTGCTTAAATTCACTCTGACATC	*Apa*I
4163	GTC TAA AAA TGT GGC TCC TCC ACT GGC GAT AAT CAG TAA TGG GTT GTC	
4164	ATC GCC AGT GGA GGA GCC ACA TTT TTA GAC GAT CCC TCG ACC TGG GAC 4165	
4165	CGCCGA GGATCC CAC AAA TAT ATT CTC CCA AGA GTC TGG C	*BamH*I
**pSVA3338 Linear fragment *Agl24*$_{up}$*-pyrEF-Agl24*$_{down}$**		
4168	ACTA GGGCCC GGTCTTGTGCTTAAATTCACTCTGACATC	*ApaI*
6336	GTA ACA TAT CTT TGC TAA ATC GGT CAT TTT CGG GTA TTA C	
4115	GTAATACCCGAAAATGACCGATTTAGCAAAGATATGTTACTCGATTACGCTAGAAAA TTTGAGCAGTTCTAGTACTTGGCTCAAAGAATG	
4116	GGT GTA CCT ATT TCG AAT TGG TTA GGT TTT CTA ACA TTT TGG ATA CTA TCT CAC TAA TTT CAT TTT TTC CTA AAA ATT GCT CCT TTA CAT TTC	
6337	GTATCCAAAATGTTAGAAAACCTAACCAATTCGAAATAGGTACACC	
6338	CGCCGA GGATCC GAA TGC CCA ATT TTT TCA TTT CTG CAA TAG TCT GTT C	*BamHI*
***S. acidocaldarius* Expression**		
pSVA1336		
4176	CTGCA CCATGGG T ATCGACAACCCATTACTGATTATCGCCAGTGG	*NcoI*
4177	CCGTT CTGCAG C TCT AAG AAA TTC TGC TAA TTC ACT TAA AAT TAT ATC	*PstII*
pSVA1339		
4180	CTGCA CCATGGG TCGACAACCCATTACTGATTATCGCCAGTGG	*NcoI*
4181	CCGTT GCGGCCGC TTA TCT AAG AAA TTC TGC TAA TTC ACT TAA AAT TAT ATC	*NotI*
E. coli* Expression of *agl24		
A596	*saci1262*-fwd-*XhoI* AGGAGA CTCGAG ATGATCGACAACCCATTACTGATTATCGCC	*XhoI*
A597	*saci1262*-rev-*KpnI* GATCCA GGTACC TCTCTAAGAAATTCTGCTAATTCACTTAAAATTATATC	*KpnI*
A684	*saci1262*_H$_{14}$A-*XhoI*-fwd AGGAGACTCGAGATGATCGACAACCCATTACTGATTATCGCCAGTGGAGGAGGGGCTACTGGCTTCGCTAGAGCTATTGC	*XhoI*
A685	*saci1262*_E$_{114}$A-fwd GTGCACTTTATGTAACAGCTAGCCAAGACAGAATTATTAC	
A686	*saci1262*_E$_{114}$A-rev GTAATAATTCTGTCTTGGCTAGCTGTTACATAAAGTGCAC	
