# Supplementary material for: Agl24 is an ancient archaeal homolog of the eukaryotic N-glycan chitobiose synthesis enzymes
Source: eLife. 2022 Apr 8;11:e67448. doi: 10.7554/eLife.67448 (PMC8993221; doi:10.7554/eLife.67448)
Supplement: Supplementary file 4. — Highlighted in grey background color are the mammalian orthologs of the non-catalytic OST subunits found in Saccharomyces cerevisiae. *based on sequence analyses of metagenome-assembled genomes of Asgardarchaeota (Zaremba-Niedzwiedzka et al.,2017), **not present in STT3 proteins from organisms that express single subunit OSTs. ***chitobiose is not present in Thermoproteales, but showing an modified version: (GlcA(NAc)2-β-1,4-Glc(NAc)2). [file elife-67448-supp4.pdf]

Domain<br>Component	**Eukarya**	**Archaea**			**Bacteria**
		Asgardarchaeota	Crenarchaeota	Euryarchaeota	
Lipid carrier	Dol	Dol	Dol	Dol	Und
Linkage between N-glycan and lipid carrier	PP	?	PP	P	PP
Nucleotide activated sugar donor	yes	?	yes	yes	yes
Lipid activated sugar donor	yes	?	yes	yes	no
Oligosaccharyltransferase	Stt3/ STT3A/STT3B	AglB (Stt3-like)	AglB (Stt3-like)	AglB (PglB-like)	PglB
DK motif (DXXKXXX(M/I)	yes	yes	yes	yes	
DKi motif (D/E< >KXXXM/I/P)				yes	
MI motif (MXXIXXX(I/V/W)				yes	yes
double sequon DNXTZNXS/T	yes**	yes	yes		
Non-catalytic OST subunits	Ost1/RPN1<br>Ost5/TMEM258<br>Swp1/RPN2<br>Wbp1/DDOST<br>Ost2/DAD1<br>Ost4 / Ost4<br>Ost6/TUSC3<br>or Ost3/MAGT1	RPN1(ribophorin-1)*<br>Ost5*<br><br>Wbp1*<br><br><br>Ost6-like/Ost3-like*	n.d.	n.d.	n.d.
First enzymes in the glycosylation	Alg7<br>Agl14/13	?	AglH<br>Agl24	diverse	PglC<br>PglA
N-glycan linking sugar(s)	Chitobiose<br>(GlcNAc- β 1,4- GlcNAc -β1-)	?	Chitobiose***	diverse	GalNAc-α1,3-*Bac*-β1
N-glycan structure	tri-branched	?	di- and tri-branched	linear, di-, tri-branched	linear
